# A retrospective study of zoonotic tuberculosis among livestock farmers of Lahore district using one health approach

**DOI:** 10.5455/javar.2024.k847

**Published:** 2024-12-27

**Authors:** Rubab Maqsood, Abdul Rehman, Farhat Nazir Awan, Hamad Bin Rashid, Shakera Sadiq Gill, Chanda Jabeen, Gulshan Umbreen, Rafia Akram, Mamoona Chaudhry

**Affiliations:** 1 Department of Epidemiology and Public Health, University of Veterinary and Animal Sciences, Lahore, Pakistan; 2 Provincial Diagnostic Laboratory, Livestock and Dairy Development, Lahore, Pakistan; 3 Department of Veterinary Surgery and Pet Sciences, University of Veterinary and Animal Sciences, Lahore, Pakistan; 4 Institute of Continuing Education and Extension, University of Veterinary and Animal Sciences, Lahore, Pakistan

**Keywords:** Zoonotic tuberculosis, bovine TB, risk factors, retrospective

## Abstract

**Objectives:**

Tuberculosis (TB) affects humans and animals regardless of species type, causing huge economic losses and deaths worldwide. However, the mechanisms and risk factors of zoonotic transmission are not well known in Pakistan. The current study aimed to identify the potential risk factors associated with TB in farmers and their animals, particularly exposure to infected animals in Lahore District, Pakistan.

**Materials and Methods:**

The study consisted of two components utilizing the concept of One Health. In the first component, a retrospective case-control study of human subjects (cases = 25, control = 25) was conducted from December 2021 to July 2022. In the second component, a cross-sectional analysis of the cattle owned by selected participants (TB cases and healthy controls) was completed in the Lahore district. A single intradermal tuberculin skin test was used to determine TB infection in cattle.

**Results:**

A total of 25 TB cases and 25 healthy controls were enrolled. Males in cases were found (OR = 0.01, 95% CI: 0.0002–0.29, *p = *0.014) less likely to get TB, cases older than 35 years (OR = 1.13 (95% CI: 1.05–1.24, *p = *0.004), unmarried cases (OR = 32.20, 95% CI: 2.92–819.03, *p = *0.014), being a smoker (OR = 21.87, 95% CI: 2.80–395.82, *p = *0.011), and keeping animals inside the home (OR = 9.92, 95% CI: 1.29–134.61, *p = *0.047) were identified as significant predictors of TB in humans in the final multivariable logistic regression. Out of 175 tested animals, 3/65 animals belonging to the cases and 1/110 animals belonging to the controls were found positive. The animals belonging to the TB cases were (OR = 7.76, 95% CI; 0.79–76.02) more likely to have a positive Single Comparative Intradermal Tuberculin Test test. The prevalence of bTB in animals belonging to the cases was 4.6% (95% CI, 1.26–12.58) compared to 0.9% (95% CI, 0.04–4.67) in animals of the control group.

**Conclusion:**

This study identified potential risk factors that could contribute to the complex web of TB transmission between humans and animals. Our findings could provide data to inform policy-making and intervention strategies to reduce TB’s burden in both populations. Embracing a holistic One Health perspective is imperative to effectively combat this shared health threat.

## Introduction

Tuberculosis (TB) has been known as a disease of poverty for decades [1] and is cited among the leading infectious causes of death in humans in the world [2]. Although *Mycobacterium tuberculosis* and *Mycobacterium bovis* are closely related members of the Mycobacterium tuberculosis complex (MTBC) and have different host preferences. Humans are assumed to be the reservoir for *M. tuberculosis* whereas *M. bovis *has a diverse host range but is usually encountered in cattle [3,4].

When TB due to *M. bovis* is communicated to humans, it is termed zoonotic TB. It is common in developing countries where bovine TB is maintained in domestic and wild animals. Transmission usually happens through inhalation of droplets and drinking of unpasteurized milk from an infected animal. Repeated isolation of *M. tuberculosis* from domestic animals accentuated its reverse zoonosis potential [5]. *Mycobacterium orygis *is also an MTBC subspecies, and it has been documented as an emergent cause of zoonotic and bovine TB in South Asia [6,7]. Recently, in Lahore, Pakistan, *M. orygis* and *M. tuberculosis* were isolated from cattle and buffalo. None of the tested animals had *M. bovis* [8]. In Lahore, Pakistan, the prevalence of MTBC was observed to be higher among occupationally exposed groups (abattoir workers, livestock farmers, animal handlers, veterinarians, and laboratory workers) [9]. Knowledge of zoonotic TB has evolved in South Asia and several other regions of the world. TB acquired from animals can be termed zoonotic TB [10].

The growing concept of One Health testified to the zoonotic blight of bovine TB. Under end TB strategy, focusing on the diagnosis and treatment of each TB patient, the policy was recently devised as a roadmap for zoonotic TB [11].

The public health system in low- and middle-income countries lacks the differential diagnostic facilities between TB of human or bovine origin due to accessibility and expensive testing techniques [12]. The top five countries with the highest burden of human TB are located in South Asia, and Pakistan is ranked fifth among these [13]. The COVID-19 pandemic has damaged, stalled, or even reversed the End TB program accomplishments, especially in high TB burden countries [14].

This study attempted to determine the risk factors that contribute to the transmission of TB from animals to humans (livestock farmers) and from TB patients to their animals in the Lahore District, Pakistan. The study also seeks knowledge, attitudes, and practices of TB patients and healthy farmers about zoonotic TB.

## Materials and Methods

### Ethical approval

Office of Research Innovation and Commercialization, University of Veterinary and Animal Sciences, Lahore, found the study per the scientific and ethical requirements and approved with reference No. DR: 56, dated 03-01-2020. Informed consent was obtained from all study participants.

### Study site

**Figure 1.**
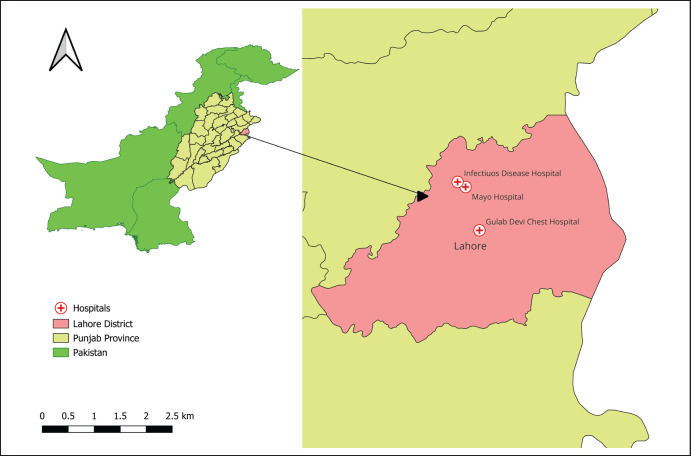
Location map of hospitals selected for study.

Lahore is a cosmopolitan city and provincial capital of Punjab with a rapidly growing population of 13 million [15]. About a century old, Mayo Hospital, Gulab Devi Chest Hospital, and Infectious Disease Hospital, located in Lahore city, were selected for the current study (Fig. 1). These hospitals receive referrals for TB and other pulmonary diseases from all over the Punjab province of Pakistan. These hospitals are also implementing the National TB Control Program under the End TB strategy of World Health Organization. Before the COVID-19 pandemic, Mayo and Gulab Devi hospitals were receiving about 40 new TB patients per day, including referrals. While at the infectious disease hospital, the average daily patient number was 5. During the COVID-19 pandemic, the count declined drastically to only 1 or 2 patients per day. During some days of lockdown, hospitals received no patients for TB. We initiated this study in December 2021, when the COVID-19 pandemic impact was waning and the average per-day patient count increased up to 10.

### Study design

Ensuring the One Health concept, a case-control study design opted to identify determinants associated with the transmission of TB between humans and their animals in the study area. This study comprised the following components:

- A case-control study of TB patients associated with livestock farming;
- Screening of animals (cattle/buffalo) belonging to TB cases and the control group of farmers;
- Knowledge attitude and practices (KAP) survey on zoonotic TB.

**Figure 2.**
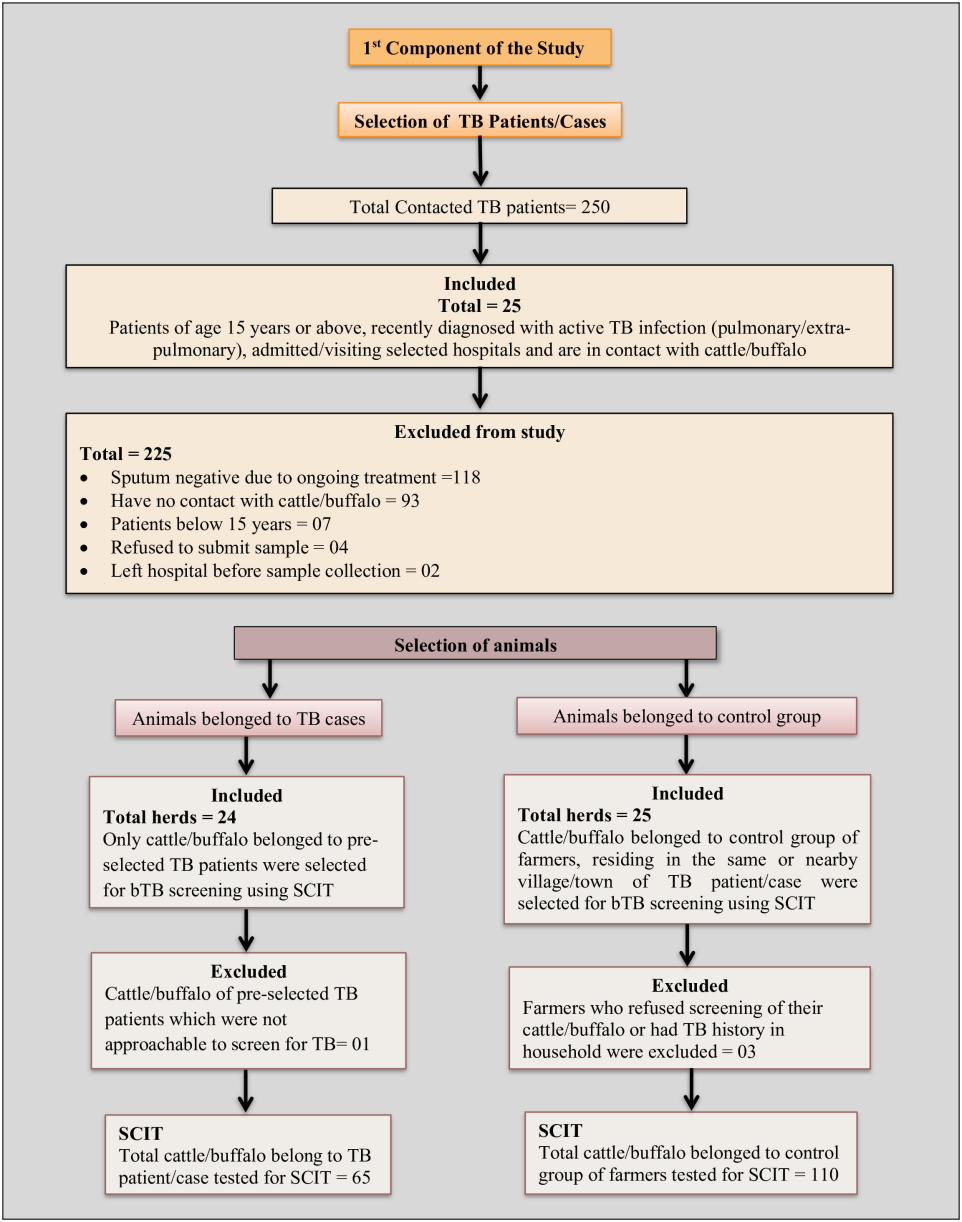
Flow diagram for study design.

TB patients admitted to TB wards of the above-mentioned hospitals were contacted from December 2021 to July 2022. The inclusion criteria for TB patients were those aged 15 years or above with active TB (pulmonary/extra-pulmonary) and in contact with cattle or buffalo, admitted to any of the selected hospitals for treatment were selected as cases for this study. The study excluded participants who were under 15 years old, had no contact with livestock, refused to provide sputum samples, or were receiving TB treatment and had a negative sputum test. Every participant in the control group was selected from the same or a nearby village or town where the case group participants were from. The control group participants were also livestock farmers with no prior history of TB diagnosis. Controls were approached with the help of respective Livestock and Dairy Development veterinarians or veterinary assistants. The livestock farmer who consented and volunteered first for sputum submission and animal screening for bovine tuberculosis (bTB) was selected from each locality (Fig. 2).

Only 25 TB patients met the inclusion criteria for cases, i.e., being 15 years old or above, positive with active TB (pulmonary/extra-pulmonary), and in contact with cattle or buffalo. All visits were made to the hospitals during the early hours of the day due to a higher influx of patients for sample submission and admission registration. Upon contacting TB patients, they were explained about the study and acquired consent. Socio-demographic, occupational, and other information related to disease symptoms were collected from all participants who gave consent and met the inclusion criteria of the study. The questionnaire was designed in English and then translated into the national language, Urdu. During formal pre-testing, it was revealed that most of the respondents were illiterate and unable to understand the questions when explained in the Urdu language. To overcome this, the Urdu format was further explicated in the local language, Punjabi.

After information was collected, participants were requested to submit a sputum sample the following morning, before eating breakfast. Hospital laboratory staff were requested to carry out the collection of extra-pulmonary samples. All samples were immediately shifted to the Disease Surveillance Laboratory Biosafety level-II in the Department of Epidemiology and Public Health, University of Veterinary and Animal Sciences Lahore, Pakistan, and stored at refrigerated temperature. Matched on a 1:1 ratio, we selected a total of 25 livestock farmers as controls. Information and sputum samples were also collected from control participants.

For the KAP survey, information was collected about knowledge, attitudes, and practices regarding zoonotic TB from all 50 participants (25 = TB cases, 25 = controls) selected for the study. This survey consisted of fourteen questions in total, with six focused on assessing knowledge and four each regarding attitudes and practices.

### Selection of risk factors

The risk factors were selected by reviewing the literature [16–18] and were categorized as socio-demographic factors (gender, age, marital status, family size, joint or nuclear families, family sizes, urban or rural residences, educational level, occupation other than livestock farming, and herd size), knowledge about zoonotic TB and livestock farming practices (can farmers get TB from animals, drinking raw milk, sharing the room with the animal at night).

### Selection of animals and testing for the second component of the study

To accomplish the second component of this study, all selected TB cases were traced to their villages/towns for tuberculin tests of their animals (cattle and/or buffalo). The animals belonging to the control group were also tested for bTB. A trained veterinarian was designated to administer tuberculin to all animals and measure skin enduration after 72 h following the guidelines of World Organization for Animal Health [19]. A total of 175 animals were subjected to a tuberculin skin test; 65 of these animals were from cases, and 110 belonged to control group members. Animal owners/workers were asked about husbandry practices (grazing, mixing with herds, history of bTB in the herd, separation of sick animals) (Fig. 2).

### Laboratory testing of human samples

All collected sputum and extra-pulmonary samples were subjected to DNA isolation for molecular detection of mycobacterium. The DNA isolation was performed using the phenol-chloroform-isoamyl alcohol and ethanol precipitation method [20]. A polymerase chain reaction (PCR) was performed to diagnose and differentiate between *M. tuberculosis, M. bovis/M. bovis *bacillus calmette-guerin (BCG), and *M. orygis.* This assay is a 3-primer PCR that detects the presence or absence of region of difference 9. A ~200 bp fragment is amplified if RD9 is present, and a ~400 bp fragment is amplified if it is absent. *M. tuberculosis *has RD9, and *M. bovis/M. bovis *BCG and *M. orygis *do not [6] (Table 1).

**Table 1.** Primer sequence.

Primers	Amplicon
RD9 primers Forward: CCGATACCATGCAACAACGG Reverse 1: CGGTCTCTCCGAGCATTC Reverse 2: GCTCGAGCTAGACCTGCAC	M. tb: 209bp Non-M. tb MTBC: 410bp

### Data analysis

Epidata version 3.1 (available via http://www.epidata.dk) was used to input the questionnaire data. This information was exported to Microsoft Excel (version 2013, Microsoft Office) for statistical processing. Validation of digital data was done by random checking for errors with the hard records. R software (version 4.2.1, R Foundation for Statistical Computing, Vienna, Austria) was used for statistical analysis. Farmers who tested positive or negative for TB were the outcome variable in all univariate and multivariate analyses. To investigate the association of various risk factors with the occurrence of zoonotic TB in humans, we conducted a case-control study involving 25 cases and 25 controls. The study employed an unmatched design. The analysis began with a univariate evaluation of each risk factor using logistic regression models, estimating the odds of zoonotic TB based on individual predictors. Variables with a *p*-value of less than 0.2 were considered for inclusion in the multivariable model.

Subsequently, a multivariable logistic regression model was constructed using a forward selection strategy. The process began with a simple model including one variable and iteratively added predictors based on theoretical relevance and statistical significance. Variables that were not statistically significant in the model were excluded before moving to the next step. This iterative process continued until the most parsimonious model was achieved, capturing the significant predictors of TB occurrence. This systematic approach ensured robustness in identifying associations while minimizing overfitting in the presence of a small sample size. The multicollinearity of predictors in the final model was assessed using the Generalized Variance Inflation Factor (GVIF). GVIF values below 5 are generally considered acceptable. After the model was fitted, the odds ratio and associated 95% confidence intervals (CIs) were calculated. The data collected about the animals’ characteristics were analyzed using Fisher’s exact test. The prevalence of bTB was calculated by using the appropriate formula [21]. For the KAP survey, percentages and crosstabulation were calculated.

## Results

### Retrospective study

In the first part of the study, 25 out of 250 contacted persons met the inclusion criteria. The control group consisted of 25 individuals. A total of 50 samples (49 sputum samples from TB cases and the control group and 01 extrapulmonary samples) were processed through PCR assays. All 25 samples (TB case group) were PCR positives for *M. tuberculosis*, but negative for *M. bovis* and *M. orygis*. Similarly, none of the sputum samples from the control group of livestock farmers was found positive for any of MTBC.

Among all study subjects (*n = *50), there were only five (10%) females (cases: 4/25, controls: 1/25) and 45 (90%) males. A total of 20 (40%) of participants (cases: 7/25, controls: 13/25) were in the age group of ≤35, while those above 35 years of age were 30 (60%), with 18 in the case and 12 in the control group. Of the participants, 40 (80%) were married, with 17 individuals (68%) in the case group and 23 (92%) in the control group; the remaining 10 (20%) were unmarried. Overall, 31 (62%) participants (cases: 19/25, controls: 14/25) were illiterate without any formal education. Livestock farming was an inherited occupation for 37 (74%) (cases: 18/25, controls: 19/25) of study subjects. About 39 (78%) farmers were living in rural (cases: 20/25, controls: 19/25), seven (14%) in peri-urban (cases: 2/25, controls: 5/25), and four (8%) in urban (cases: 3/25, controls: 1/25) settings (Table 2.).

The average monthly earnings of 33 (66%) participating farmers were ≤30 thousand Pakistani rupees (PKR)/- with a disproportionate distribution among cases (84%, 21/25) and controls (48%, 12/25). Of the participants, 41 (82%) lived in joint family settings, with a comparable distribution to the cases (80%, 20/25) and control farmers (84%, 21/25). Family size of 31 (62%) study participants consisted of >6 individuals (cases: 64%, controls: 60%). The home unit consisted of ≤2 rooms according to 29 (58%) (cases: 17/25, controls: 14/25) participants. Regarding the childhood BCG vaccine, 32 (64 %) (cases: 10/25, controls: 22/25) of subjects responded having been vaccinated, while 16 (32%) (cases: 13/25, controls: 2/25) were non-vaccinated, and two (4%) did not know about their vaccination status. Among the cases, 19 (76%) were smokers, compared to 13 (44% ) in the control group. Compared to the cases (8%, 2/25), the control group had a higher rate of meat availability (three times per week; 24%, 6/25). Four cases had chronic conditions (HIV: 1/25, Hepatitis: 1/25, Diabetes: 2/25) other than TB, while three control individuals also reported having diabetes and hypertension, among other chronic ailments (Table 2).

**Table 2.** Univariable analysis of socio-demographic factors of the TB case and control selected for the study in Lahore.

Factors	Cases/TB patients (*n* = 25) (%)	Controls/Healthy farmers (*n* = 25) (%)	Total	*p*-value (> 0.05)	OR (95%CI)
Gender					
Male	20 (80)	24 (96)	44	0.115	–
Female	5 (20)	1 (4)	6		
Age					
≤35	7 (28)	13 (52)	21	0.070	–
>35	18 (72)	12 (48)	29		
Marital status					
Unmarried	8 (32)	2 (8)	10	0.047 *	5.4 (1.18–39.00)
Married	17 (68)	23 (92)	40		
Education					
Illiterate	19 (76)	12 (48)	31	0.045 *	3.4 (1.06–12.16)
literate	6 (24)	13 (52)	19		
livestock farming as an inherited occupation					
Yes	18 (72)	19 (76)	37	0.747	–
No	7 (28)	6 (24)	13		
Years of farming experience if livestock farming is not an inherited occupation					
≤5 years	3 (42.8)	3 (50)	6	0.797	–
Above 5 years	4 (57.2)	3 (50)	7		
Residence					
Rural	20 (80)	19 (76)	39	0.158	–
Urban	3 (12)	1 (4)	4		
Peri-urban	2 (8)	5 (20)	7		
Monthly income					
≤30 thousand PKR/-	21 (84)	12 (48)	33	0.010 *	5.69 (1.61–23.98)
>30 thousand PKR/-	4 (16)	13 (52)	17		
Family type					
Nuclear	5 (20)	4 (16)	9	0.713	–
Joint	20 (80)	21 (84)	41		
Family size					
≤6	9 (36)	10 (40)	19	0.771	–
>6	16 (64)	15 (60)	31		
Number of rooms in the house					
≤2	17 (60)	14 (56)	29	0.384	–
>2	8 (40)	11 (44)	21		
Vaccinated (BCG) against TB in childhood					
Yes	10 (40)	22 (88)	32	0.995	–
No	2 (8)	0	2		
Don’t know	13 (52)	3 (12)	16		
Smoker					
Yes	19 (76)	11 (44)	30	0.024 *	4.03 (1.25–14.37)
No	6 (24)	14 (56)	20		
Meat consumption per week					
Once and twice	23 (92)	19 (76)	17	–	–
Thrice or more	2 (8)	6 (24)	8		
Chronic illness other than TB					
Yes	4 (16)	3 (12)	7	–	–
No	19 (76)	21 (84)	40		
Don’t know	2 (8)	1 (4)	3		
Do any of the family members or persons in contact have/had TB?					
Yes	9 (36)	2 (8)	10	0.027	6.47 (1.43–46.31)
No	16 (64)	23 (92)	40		
Diagnosed/suffered from TB before this time?					
Yes	1 (4)	0	1	–	–
No	24 (96)	25 (100)	49		
Animal keeping place					
Inside home	10 (40)	2 (8)	12	0.015 *	7.67 (1.72–54.64)
Outside home	15 (60)	23 (92)	38		

* p-value < 0.05.

Among selected participants of the study, nine (36%) TB cases were previously in contact with TB patients in their family/friends, whereas only two (8%) persons in the control group reported contact with TB patients in their family/friends. One individual in the TB case group was suffering from a reoccurrence of TB.

### Univariable analysis results for retrospective study on TB cases and controls

In the univariable analysis, several variables demonstrated significant associations with TB in occurrence in cases. Odds ratios greater than one, indicated an increased likelihood of disease occurrence in cases with the presence of these factors. Marital status was significantly associated with TB in cases. Unmarried individuals in the case group were 5.41 (95% CI: 1.18–39.00, *p = *0.047) times more likely to get TB than controls. Education level also showed a significant association, with illiterate participants in cases having a 3.43 (95% CI: 1.06–12.16, *p = *0.045) times higher likelihood of TB than controls. Livestock farmers in the case group with a monthly income of ≤30,000 PKR were 5.69 (95% CI: 1.61–23.98, *p = *0.010) times more likely to have TB compared to those earning more than 30,000 PKR. Cases having a history of contact with TB patients in family/friends were 6.47 (95% CI: 1.43–46.31, *p = *0.027) more likely to have TB than controls. Smokers in the TB case group had times 4.03 (95% CI: 1.25, 14.37, *p = *0.024) higher likelihood of having TB among cases compared to non-smokers. Livestock farmers with TB were 7.67 (95% CI: 1.72–54.64, *p = *0.047) times more likely to keep animals inside the home compared to farmers without TB (controls) (Table 2).

### Multivariable analysis

The multivariable logistic regression model identified five significant risk factors for TB among cases. Males in the cases group had substantially lower odds of contracting TB than females, with an odds ratio of 0.01 (95% CI: 0.0002–0.29, *p = *0.014). Participants with ages more than 35 years in the case group were 1.13 (95% CI: 1.05–1.24, *p = *0.004) times more likely to have TB compared to the healthy livestock farmers (controls). Unmarried individuals in the cases were 32.20 (95% CI: 2.92–819.03, *p = *0.014) times more likely to have TB compared to the controls. Smokers in the case group had 21.87 (95% CI: 2.80–395.82, *p = *0.011) times higher likelihood of TB than non-smokers. Livestock farmers with TB were 9.92 times (95% CI: 1.29–134.61, *p = *0.047) more likely to keep animals inside homes than the control (Table 3).

Multicollinearity was checked, and all predictors had GVIF values below 2, with their corresponding adjusted values (GVIF^(1/(2*df))) ranging from 1.10 to 1.42. These results indicate low multicollinearity among the variables included in the model, as GVIF values below 2 indicate minimal concern. Therefore, the stability of the regression coefficients is unlikely to be compromised by collinearity, supporting the reliability of the model’s estimates.

**Table 3.** Multivariable analysis of the information collected from the TB case and controls from selected hospitals in Lahore.

No.	Variables	Response	OR	CI (95%)	*p*-value < 0.05
1	Gender	Male	0.01	(0.0002–0.29)	0.014
		Female			
2	Age	≤35	1.13	(1.05–1.24)	0.004
		>35			
3	Marital status	Unmarried	32.20	(2.92–819.03)	0.014
		Married			
4	Smoker	Yes	21.87	(2.80–395.82)	0.011
		No			
5	Animal keeping place	Inside home	9.92	(1.29–134.61)	0.047
		Outside home			

Wider CIs observed in our model likely resulted from a smaller sample size. Further studies with balanced and larger sample sizes can increase the precision.

### Characteristics of the TB cases enrolled in the study from the selected TB hospitals

According to the patient history file, one out of the 25 TB cases had a diagnosis of rifampicin-resistant TB. Seven (28%) TB cases had unsatisfactory health status. Chronic cough (96%, 24/25) for more than 3 weeks, fever (84%, (21/25), pain in the chest (88%, 22/25), coughing up blood or sputum (36%, 9/25), weakness or fatigue (96%, 24/25), weight loss (84%, 21/25), sweating at night (40%, 10/25), and diarrhea (36%, 9/25) were the main clinical indications that TB patients narrated (Table 4).

### Prevalence of bTB in animals

A total of 172 animals were tested for bTB using the single comparative intradermal tuberculin test (SCIT) test. The number of animals belonging to TB cases was smaller (65) than the number of animals (110) in the control group. There were 3 animals in the case group and one in the control group that tested positive for SCIT. The prevalence of bTB in animals belonging to the case group was 4.6% (95% CI, 1.26–12.58) compared to 0.9% (95% CI, 0.04–4.67) in animals of the control group. Overall prevalence among a total of 175 tested animals was 2.28% (95% CI, 0.77–5.53). The results suggest a higher prevalence of bTB among animals belonging to the TB case group than the animals of the control group.

### Risk factors for SCIT-positive test results

The animals belonging to the TB case group were 7.76 (95% CI; 0.79–76.02) times more likely to have positive SCIT tests compared to the animals belonging to the control group of livestock farmers. Animal age, source of animal in the herd, animal milking status, animal pregnancy, and gestation number showed no association with the SCIT-positive status of all the tested animals (Table 5). Further studies with larger sample sizes and molecular diagnostic techniques are needed to draw a definitive conclusion.

**Table 4.** Characteristics of the TB cases enrolled in the study from the selected TB hospitals in Lahore.

Factors	TB cases response	%
Type of TB identified		
Drug sensitive	24	96
Drug-resistant	1	4
Perceived health status according to TB patient		
Satisfactory	18	72
Unsatisfactory	7	28
Chronic cough for more than the last 3 weeks?		
Yes	24	96
No	1	4
Since you feel sick do you have a fever?		
Yes	21	84
No	4	16
Pain in the chest		
Yes	22	88
No	3	12
Coughing up blood or sputum		
Yes	9	36
No	16	64
Weakness or fatigue		
Yes	24	96
No	1	4
Weight loss		
Yes	21	84
No	4	16
Sweating at night		
Yes	10	40
No	15	60
Diarrhea		
Yes	9	36
No	16	64

### KAP survey

Out of 50 farmers surveyed (25 = cases, 25 = controls), 6 (12%) responded that TB affects animals, 25 (50%) said that TB does not affect animals, and 19 (38%) stated that they were unaware of any impact of TB on animals. Of all, 23 (46%) said they cannot get TB from animals; 22 (44%) said they do not know, and 5 (10%) said they can get it from animals. Cough spray can transmit TB, according to 37 (74%) study participants, nine (18%) individuals said no, and four (8%) responded that they did not know. About 20 (80%) of the TB cases were not wearing face masks during coughing and talking to their attendants. Farmers’ response to raw milk consumption and transmission of TB from animals to humans could not be analyzed due to zero cell value in cross tabulation. Of the 50, 35 (70%) (No = 06, ofte*n = *09) stated that they wash their hands with soap after handling animals. Most of the farmers (41; 82%) were offering green fodder to the animals as feed (Table 6). No significant association was found for actors included in the KAP survey.

**Table 5.** Risk factors analysis for animals belonging to TB cases and controls.

No.	Variables	OR (95%CI)	*p*-value < 0.05
1	Animal belonging to	7.76 (0.79–76.02)	0.039
	TB case group		
	Control group		
2	Animal age	2.17 (0.17–116.03)	0.86
	1–5 years		
	>5 years		
3	Source of animal in the herd	0.76 (0.10–5.54)	0.79
	Animal purchased from another herd		
	Raised in the same herd		
4	Animal milking status	0.61 (0.06–6.10)	0.67
	Yes		
	No		

**Table 6.** Knowledge attitude and practices of TB case and control livestock farmers about zoonotic TB in Lahore.

Knowledge attitude and practices	TB cases (*n* = 25) (%)	Controls (healthy farmers) (*n* = 25) (%)	Total
Can TB affect animals?			
Yes	4 (16)	2 (8)	6
No	13 (52)	12 (48)	25
Don’t know	8 (32)	11 (44)	19
Can you get TB from animals?			
Yes	4 (16)	1 (4)	5
No	13 (52)	10 (40)	23
Don’t know	8 (32)	14 (56)	22
Can cough spray transmit TB?			
Yes	19 (76)	18 (72)	37
No	5 (20)	4 (16)	9
Don’t know	1 (4)	3 (12)	4
Use of facemask or cover face while coughing?			
Yes	3 (12)	7 (28)	10
No	20 (80)	7 (28)	27
Some times	2 (8)	11 (44)	13
Do you think, drinking raw milk can transmit TB?			
Yes	8 (32)	10 (40)	18
No	14 (56)	15 (60)	29
Don’t know	3 (12)	0	3
Sharing a room with animals can spread TB?			
Yes	1 (4)	2 (8)	3
No	14 (56)	12 (48)	26
Don’t know	10 (40)	11 (44)	21
Do you consume unpasteurized/raw milk?			
Yes	2 (8)	0	2
No	15 (60)	22 (88)	37
Both	8 (32)	3 (12)	11
Do you wash your hands with soap after working with animals?			
Always	17 (68)	18 (72)	35
No	5 (20)	1 (4)	6
Often	3 (12)	6 (24)	9
House/shed sharing with animals at night or sleeping in pen			
Yes	21 (84)	15 (60)	36
No	4 (16)	10 (40)	14
Separation of sick animals from the rest of the herd			
Yes	1 (4)	4 (16)	5
No	24 (96)	21 (84)	45
Presence of other livestock among bovines			
Yes	16 (64)	14 (56)	30
No	9 (36)	11 (44)	20
Grazing			
Yes	11 (44)	7 (28)	18
No	14 (56)	18 (72)	32
If grazing yes then mixing of animals with other herds during grazing and watering			
Yes	10 (90.91)	4 (57.15)	14
No	1 (9.09)	3 (42.85)	4
Feed offered to animals			
Green fodder	21 (84)	20 (80)	41
Green fodder + concentrate	4 (16)	5 (20)	9

## Discussion

In Pakistan, *M. bovis* has been reported as the main cause of zoonotic and bTB [22]. Recent studies from Pakistan and South Asia highlighted the changing landscape of bovine and human TB [6,8]. Keeping that in view, the present study aimed to identify the risk factors contributing to TB transmission between animals and humans, specifically among livestock farmers, and from TB patients to their animals in the Lahore District, Pakistan.

The final multivariable logistic regression model identified that males in the cases group were substantially less likely (OR = 0.01, *p = *0.014) to contract TB than females. It has been reported that females were 2.06 times more likely to contract TB in Pakistan [23]. A higher prevalence of TB in females compared to males has been reported in Khyber Pakhtunkhaw, Bajaur and Islamabad, Pakistan [24-26]. Although other studies from different countries reported higher odds ratios and prevalence of TB in males [27,28]. Participants with ages more than 35 years in the case group were more (OR = 1.13, *p = *0.004) likely to have TB compared to the healthy controls. The highest TB notification rates worldwide occur among those aged 45–55, with a marked increase in the age group of >65 years [29,30]. There could be some underlying factors like age-related changes in immunity, comorbidity, and lifestyle that can contribute to the development of TB [31].

Unmarried individuals in the cases were (OR = 32.20, *p = *0.014) more likely to have TB compared to the controls. TB is viewed as a social stigma in Pakistan; TB patients of both genders perceive this disease can compromise their marriageability [32]. In some other countries in South Asia, TB has been a social stigma and a hurdle in getting married and also impacts married life [33]. A study from Pakistan reported that subjects with single marital status are 11.1 times more likely to have multi-drug-resistant TB [34]. Smokers in the case group had (OR = 21.87, *p = *0.011) a higher likelihood of TB than non-smokers. A study in Indonesia reported that smokers are 3.34 times more likely to get TB [35]. Smokers are twice as likely to develop TB than nonsmokers. Smoking over time can alter lung function, increasing the risk of developing TB [36]. Livestock farmers with TB were (OR = 9.92, *p = *0.047) more likely to keep animals inside their homes than the control group. Univariable analysis showed that TB cases had less income (OR = 5.69, *p = *0.010) than controls. They might not have enough money and land to build a separate animal shed.

Data analysis showed that the animals belonging to TB cases were 7.76 (*p = *0.039) times more likely to have a positive tuberculin test than the animals belonging to the control group. It has been reported that cattle owned by TB farmers had three times higher tuberculin positivity compared to cattle owned by farmers without active TB [37]. The presence of TB patients and keeping animals inside the household was reported as a risk factor for animals getting infected with *M. tuberculosis* [38]. Different studies have documented that cattle can contract *M. tuberculosis* from farmers and farm workers due to close contact [39–41]. A recent study on cattle and buffalos slaughtered in a slaughterhouse in Lahore reported *M. orygis *and *M. tuberculosis* as the primary cause of bTB. No animal was found positive for *M. bovis *[8].

In this study, differential PCR testing showed that none of the TB cases were found positive for *M. bovis *or *M. orygis*. Keeping in view all statistical results, TB among cases due to *M. tuberculosis *might have occurred due to other predisposing factors. Though spillover of *M. tuberculosis* from active TB cases to their animals could be a plausible event, this study could not provide strong evidence for direct transmission of TB from the cases to their animals and vice versa.

Observed symptoms among TB cases were cough for more than 3 weeks (96%), fatigue (96%), fever, and weight loss (84%), which were aligned with the commonly reported symptoms associated with pulmonary TB [42].

This study’s cases were only selected from government hospitals, which might not be representative, as people with high socioeconomic status prefer to visit private hospitals and clinics. Other limitations of this study are the small sample size and unmeasured confounding. Contrary to the human subjects in the study, we had to rely only on tuberculin tests to diagnose TB in animals.

## Conclusion

Gender, age, marital status, illiteracy, low income, smoking, and keeping animals indoors were significant risk factors for TB among farmers, suggesting socioeconomic status plays a crucial role in TB transmission. While no evidence of zoonotic transmission was found, high tuberculin positivity in animals exposed to TB patients raises concerns about potential reverse zoonosis. A study with an increased sample size using molecular diagnosis in both animals and humans can help in a better understanding of the transmission of TB in humans and animals and their relatedness to the host. The study also underscores the role of livestock management practices, such as keeping animals inside households, as a potential risk factor for TB transmission. These findings emphasize the need for integrated human-animal health programs to address zoonotic TB.

## List of abbreviations

BCG, Bacillus Calmette-Guerin; bTB, Bovine Tuberculosis; CI, Confidence interval; GVIF, Generalized Variance Inflation Factor; KAP, Knowledge Attitude and Practices; MTBC, Mycobacterium tuberculosis complex; PCR, Polymerase Chain Reaction; OR, Odds Ratio; SCIT, Single Comparative Intradermal Tuberculin Test; TB, Tuberculosis; WHO, World Health Organization.
